# No-reflow in acute ischemic stroke: mechanisms, measurements, and molecular markers

**DOI:** 10.3389/fneur.2026.1832719

**Published:** 2026-09-01

**Authors:** H. Bayona, M. F. Granja, J. N. Useche

**Affiliations:** 1 Neurology Service, Fundacion Valle del Lili, Cali, Colombia; 2 Department of Radiology, LaCardio, Bogotá, Colombia; 3 Department of Radiology, Hospital Universitario Mayor Mederi, Bogotá, Colombia; 4 Clinical Radiology & Imaging Sciences, Indiana University School of Medicine, Indianapolis, IN, United States

**Keywords:** no-reflow phenomenon, reperfusion, stroke, thrombectomy, thrombolytic therapy

## Abstract

**Background:**

No-reflow is a critical microvascular phenomenon in acute ischemic stroke in which patients fail to achieve adequate tissue reperfusion despite successful recanalization of a large vessel occlusion. It affects at least 25% of patients after reperfusion and results from persistent microvascular dysfunction that prevents restoration of normal blood flow to ischemic tissue, leading to ongoing injury.

**Objective:**

This paper synthesizes historical, mechanistic, diagnostic, and therapeutic perspectives on the no-reflow phenomenon to clarify its pathophysiology and highlight gaps limiting clinical translation.

**Methods:**

A narrative review was conducted using foundational experimental studies and contemporary clinical literature. Evidence was organized into mechanistic domains, measurement modalities, and therapeutic strategies, integrating molecular, imaging, and neurointerventional findings.

**Results:**

No-reflow arises from interacting mechanisms, including microvascular occlusions from embolic fragments, platelet aggregation, erythrocyte/fibrin deposition, and leukocyte plugging, where neutrophils can lodge within capillaries and impede flow. Vascular remodeling driven by endothelial dysfunction, oxidative stress, necroptosis, microglial activation, and pericyte constriction further compromises perfusion, while cytotoxic edema produces microvessel compression. Measurement remains inconsistent due to the absence of standardized diagnostic criteria, with current tools ranging from TICI-based angiographic assessments to perfusion imaging and molecular biomarkers. Emerging therapies target distal embolization, microvascular thrombosis, vasoconstriction, inflammation, and ischemic conditioning, while selective hypothermia shows promise in experimental models.

**Conclusion:**

No-reflow phenomenon is a multifactorial barrier to functional recovery after stroke, and its clinical impact is amplified by diagnostic ambiguity. Advancing real-time measurement tools and mechanism-based therapies is essential to improving outcomes after recanalization.

## Introduction

No-reflow is a critical phenomenon in acute ischemic stroke where, despite successful revascularization of a large vessel occlusion (LVO), patients fail to regain functional independence due to microvascular dysfunction (1). This paradoxical situation prevents normal blood flow to brain tissues, leading to persistent tissue damage (1). This phenomenon happens in at least 25% of the cases after reperfusion (2, 3), but varies depending on diagnostic criteria and is 1 to 81% of reported data (4–6). The “no-reflow” refers to distal hypoperfusion that occurs despite advancements in thrombolysis and thrombectomy, which facilitate proximal large artery recanalization in AIS patients (4). It primarily affects precapillary arterioles, capillaries, and postcapillary venules (7), which are microvessels measuring between 6 and 20 μm and composed of endothelial cells, astrocytes, microglia, neurons, and pericytes (1, 2). Scientific inquiry over half a century has validated its existence in both animal models and human subjects, and its correlation with adverse clinical outcomes underscores the need to address it for enhancing AIS prognosis (4). It has been described in other organs like heart (8), skeletal muscle and kidney (7). There is no synonymy between no reflow and futile reperfusion or clinically ineffective reperfusion.

## Historical aspects of no reflow

The concept of cerebral “no-reflow” first reported in 1960 (7) and then corroborated from groundbreaking studies by Ames et al. (9), who observed that parts of the rabbit brain did not regain perfusion after restoring proximal large artery flow following global cerebral ischemia. Initial research suggested that blood components trapped within capillaries contributed to hypoperfusion, and later studies highlighted the crucial role of blood components in “no-reflow”. Subsequent research, including work by Crowell and Olsson in 1972 on focal ischemic models (10), and Little et al. (11), further identified microvascular filling impairments and the role of swollen endothelial cells and astrocyte endfeet in constricting microvessels. Clinical evidence for cerebral “no-reflow” first emerged in 1994 when Baird et al. detected it in a patient treated with streptokinase for thrombolysis, observing a mismatch between successful large artery recanalization and reperfusion failure using single-photon emission computed tomography (SPECT). The application of magnetic resonance perfusion (MRP) technology by Albers et al. in 2006 further pioneered the identification of “no-reflow” in patients post-thrombolysis (12).

## Mechanisms of no-reflow

**Figure 1:**
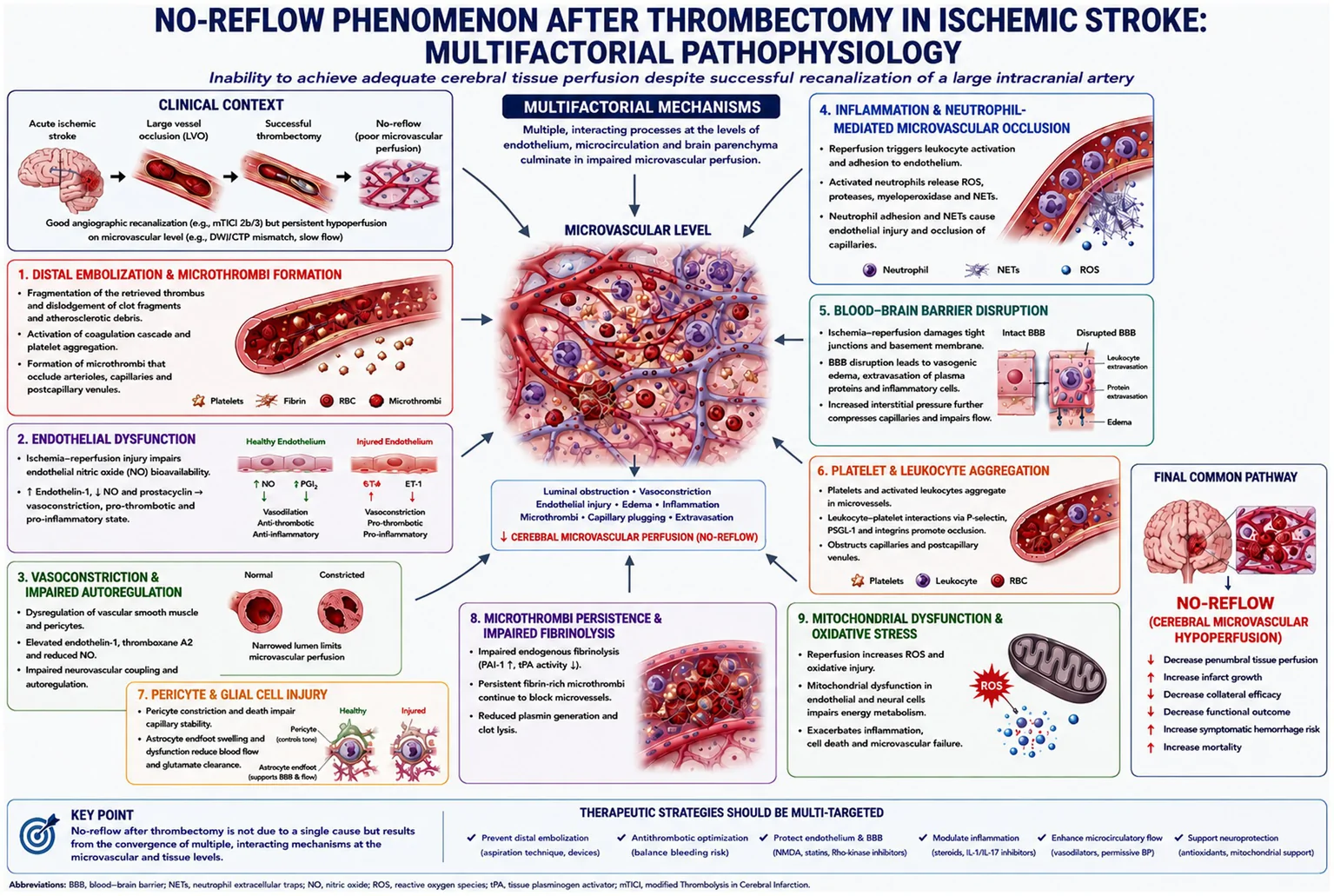
Summary of the phenomenon.

Several mechanisms contribute to the no-reflow phenomenon, often involving the dysfunction of various cellular components within the brain’s vasculature (1). The “no-reflow” phenomenon is multifaceted, involving microemboli formation, microvascular compression, and microvessel contraction (4, 7) (Figure 1).

## Microvascular occlusions

Embolic fragments from the original thrombus or local thrombus formed by *in situ* platelet activation, adherence and aggregation can occlude microvessels. Erythrocytes and fibrin deposition are other contributing factors in diminishing microcirculation (1, 7).

Neutrophils and other leukocytes can become lodged within the capillary network, impeding blood flow and worsening tissue damage. This stalling has been observed to cause stagnant cerebral blood flow, and anti-neutrophil therapies have shown promise in restoring microvascular perfusion. Retrieval of an LVO can dislodge clots, sending them downstream to cause distal occlusions. Softer clots, often rich in erythrocytes, are more prone to migration, while stiffer, platelet- and fibrin-rich clots are less likely to migrate.

## Vascular remodeling with microvessel compression and or contraction

Rapid loss of cerebral blood flow in ischemic stroke leads to immediate detrimental vascular remodeling, partly due to the inhibition of endothelial NO synthase (eNOS). Inflammation and ROS exacerbate endothelial damage, necroptosis (2), microglia activation, and pericyte dysfunction (4). Disruption of gap junctions between endothelial cells (ECs) and smooth muscle cells, crucial for maintaining proper vascular tone, also contributes to dysregulated vascular tone. Pericytes, highly sensitive to ischemia (7), can undergo irreversible constriction and eventual cell death, further impeding blood flow. Pericyte contraction due to oxidative-nitrative stress and calcium overload. Astrocytes also play a role in regulating cerebral blood flow, and their dysfunction during stroke can lead to vasoconstriction (1, 7).

Microglia transition to a reactive phenotype after stroke, contributing to blood–brain barrier breakdown and releasing proteases that further damage vessels. Microvascular compression is driven by cytotoxic edema, particularly due to astrocytic endfeet swelling which is mediated by ion channels and aquaporins.

## Measurements of no-reflow

Currently there are no definitions or diagnostic criteria for no reflow (1), and there is a paucity of agreement in the measurement using diagnostic techniques (4). Accurate and consistent measurements for no-reflow remain a challenge, with various approaches being explored (Table 1).

**Table 1:** Comprehensive overview of no-reflow in acute ischemic stroke (18).

Category	Factor	Description/role	Limitations/notes	Evidence/references
Mechanisms	Microvascular occlusions	Embolic fragments or local thrombus occlude microvessels; distal embolization during thrombectomy.	Observed in clinical and animal studies; clot composition influences migration.	Goyal et al., *Stroke*, 2016 (19)
	Vascular remodeling	eNOS inhibition reduces vasodilation; pericyte constriction; astrocyte and microglial dysfunction disrupt vascular tone and BBB.	Immediate after ischemia; irreversible pericyte constriction documented in models.	del Zoppo et al., *Stroke*, 1991 (20)
	Stalled neutrophils	Neutrophils lodge in capillaries, causing stagnant flow; anti-neutrophil therapy restores perfusion in models.	Mainly experimental evidence; clinical translation ongoing.	Bui et al., *Frontiers in Aging*, 2022 (21)
Measurements	TICI/mTICI (angiography)	Assesses reperfusion via capillary blush during digital subtraction angiography.	Inconsistent definitions; macrovascular focus, not microvascular.	Kallmes et al., *J NeuroIntervent Surg*, 2018 (9)
	Modified Rankin Scale (mRS)	Functional outcome score; mRS ≥ 3 used as proxy for futile reperfusion.	Indirect measure; influenced by multiple factors beyond microvascular perfusion.	Hussein et al., *Neuroradiology*, 2018 (22)
	Perfusion CT/MRI	Evaluates mean transit time and variance to infer microvascular function.	Limited resolution for capillary-level changes; advanced imaging required.	Campbell et al., *Curr Neurol Neurosci Rep*, 2019 (23)
	LSCI & Two-photon microscopy	Visualizes cerebral blood flow and capillary patency in animal models.	Experimental; not clinically applicable yet.	Sullender et al., *Neurophotonics*, 2018 (24)
	Histopathology (*Ex Vivo*)	Staining and microscopic analysis of vessel patency post-recanalization.	Requires tissue samples; not clinically practical.	McBride et al., *Translational Stroke Research, 2018* (25)
Molecular markers	Neutrophil count & NLR	High neutrophil accumulation and elevated NLR correlate with poor reperfusion.	Prognostic marker; linked to inflammation and microvascular plugging.	Semerano et al., *Stroke*, 2019 (26)
	Interleukin-6 (IL-6)	Elevated IL-6 linked to no-reflow; low IL-6 predicts complete reperfusion.	Reflects systemic inflammation; dynamic changes during stroke.	Lamberset et al., *Acta Neuropathol*, 2019 (27)
	Interleukin-1 (IL-1)	Associated with impaired reperfusion and inflammatory cascade activation.	Target for anti-inflammatory therapies.	Planas et al., *Stroke*, 2018 (28)
	GFAP, Tau, Neurofilament light chain	Increased levels indicate neuronal and glial injury; correlate with poor outcomes.	Biomarkers of tissue damage rather than direct perfusion.	Gaetani et al., *J of Neurology, Neurosurgery & Psychiatry* 2019 (29)
	CXCL-1, 12	Elevated in occlusion region; acts as T-cell chemoattractant and prognostic indicator.	Experimental and translational evidence.	Zhang et al., Front. Immunol., 2021 (30)
	P- and E-Selectins	Upregulated in cerebral microvasculature and peripheral blood; mediate leukocyte adhesion.	Potential therapeutic targets for reducing leukocyte plugging.	Planas et al., *Stroke*, 2018 (28)
	Egr-1	Knockout models show improved postischemic flow, implicating Egr-1 in no-reflow.	Genetic evidence; preclinical models only (18).	Yan et al., *Nature Medicine*, 2000 (31)
	High blood glucose	Hyperglycemia associated with poor reperfusion and worse outcomes.	Common clinical risk factor; modifiable.	Martini et al., *J Cereb Blood Flow Metab*, 2008 (18)

Source: author elaboration. TICI, thrombolysis in cerebral infarction; NLR, neutrophil count and neutrophil-to-lymphocyte ratio; GFAP, glial fibrillary acidic protein; CXCL, C-X-C motif chemokine ligand.

### Neurointerventional measurements

The Thrombolysis in Cerebral Infarction (TICI) scale is used to assess reperfusion by measuring capillary blush during digital subtraction angiography (DSA) (1). However, definitions for successful recanalization and reperfusion using TICI/mTICI scores vary across studies, leading to inconsistencies (9). Extended modified TICI scale eTICI is the score used in clinical studies of thrombectomy and intervention trials and inclusion of TICI2b, 2c could overestimate the no-reflow phenomenon (13). Probably TICI score based on DSA could be sooner in time for correct diagnosis of no-reflow. Where recanalizacion IIb-III happens in 92.8% according to the study of a Spain series (14). The hypoperfusion pattern appears in 100% of TICI 2a, 63% 2b and 43% in TICI III. With a good correlation between NIHSS and hypoperfusion volume with r = 0.38 (*p* < 0.01). Some studies use modified Rankin Scale (mRS) scores as a proxy for no-reflow, classifying scores of at least 3 as evidence of futile reperfusion, though this approach has limitations as mRS can be influenced by multiple factors.

### Neuroimaging

From colloidal carbon staining to modern perfusion imaging (CTP, MRP, Arterial Spin Labeling ASL) (8). Techniques like perfusion computed tomography (CT) and magnetic resonance imaging (MRI) (3) are used to assess mean transit time and its variance, which can indicate microvascular functionality. Intravoxel incoherent motion (IVIM) imaging also shows a reduction in perfusion fraction within both the ischemic core and the penumbral region, which may serve as a marker of microvascular impairment compared with healthy brain tissue and is associated with poor prognosis (15). BOLD (blood oxygen level–dependent) MRI reflects cerebrovascular reactivity (16), and delayed responses in regions with poor reperfusion indicate underlying microvascular damage. In addition to the modalities discussed, recent studies have shown that fluid attenuation recovery (FLAIR)- hyperintense vessels (FHV), which reflect slow flow within the cortical arteries, are associated with impaired collateral circulation. This study (17) evaluated its presence after acute ischemic stroke and successful thrombectomy and found that it is a robust imaging biomarker of persistent perfusion deficit and early worse neurologic outcome (poor NIHSS shift after thrombectomy), and decreased likelihood of hemorrhagic transformation. The authors hypothesized that this impaired flow could reflect the no-reflow phenomenon and could serve as a non-invasive, useful biomarker (17). TCD offers real-time microvascular resistance monitoring using pulsatility index (PI) or resistance index (RI). ASL is another method without exposure of contrast media with good spatial and temporal resolution (4).

Laser speckle contrast imaging (LSCI) and 2-photon microscopy are used in animal models to monitor cerebral blood flow and visualize microvascular networks, assessing capillary patency and red blood cell velocity. Also contrast-enhanced ultrasound, near infrared spectroscopy (NIRS), and diffuse correlation spectroscopy (DCS) for microcirculation mapping are being used.

## Histopathologic measurements

Ex vivo histological studies are used to determine vessel patency after recanalization, often involving staining for microvascular patency (Table 1). These methods provide insight into microvascular architecture but are limited in clinical translation due to the need for biopsy (1).

## Molecular markers of no-reflow

Several molecular markers have been identified as potential prognostic indicators for no-reflow (1) (Table 1).

### Neutrophil count and neutrophil-to-lymphocyte ratio

Increased neutrophil accumulation and high NLR are associated with poor clinical outcomes and poor reperfusion. Neutrophils contribute to cerebral ischemia–reperfusion injury by increasing rolling and adhesion in venules.

### Interleukins

High levels of interleukin-6 (IL-6) are associated with no-reflow, and low levels upon admission are linked to complete reperfusion. IL-6 is involved in inflammation, thrombosis, and hemostasis. Interleukin-1 (IL-1) levels have also been shown to predict reperfusion efficacy (1).

## Other markers

Elevated rates of change in glial fibrillary acidic protein (GFAP), total-tau, and neurofilament light chain are associated with poor clinical outcomes. The T cell chemoattractant CXCL-11 (C-X-C motif chemokine ligand 11) is significantly elevated in the region of occlusion and is a potential prognostic indicator. P- and E-selectins (2) are elevated in both cerebral microvasculature and peripheral blood in stroke models.

Early growth response gene-1 (Egr-1) knockout mice show increased postischemic cerebral blood flow, suggesting its role in no-reflow. High blood glucose is also associated with poor clinical outcomes (1, 2, 7).

## Treatment strategies

Reducing recanalization times theoretically reduces possibility of no-reflow because of the results in animal models and in myocardial infarction.

Prevention of distal embolization: Strategies such as embolic protection devices, thrombus aspiration, and intra-arterial thrombolysis can reduce clot fragmentation and improve reperfusion (4, 7).

Shortened Onset to Recanalization Time: reducing the time from stroke onset to recanalization can decrease the incidence of no-reflow, as prolonged ischemia is linked to its development (4, 7).

Antithrombotic and Antiplatelet Therapies: These therapies, including GP IIb/IIIa receptor antagonists, phosphodiesterase (PDE) inhibitors, can prevent microvascular thrombosis and improve blood flow. Cilostazol and glycoprotein IIb/IIIa agents used during reperfusion therapies are an add on therapy. Von Willebrand factor (vWF) (2) inhibitors by blocking glycoprotein Ibα or using ADAMTS13 cleaving vWF, gene-editing therapies (4, 7).

Vasodilators: Therapeutic approaches that prevent pericyte constriction or astrocyte swelling, and agents like adenosine and nitric oxide, can reduce cerebral capillary resistance and restore microcirculation patency. Compression-targeting: Mannitol, hypothermia, AQP4 modulation, nanoparticles. Contraction-targeting: Nimodipine, nicardipine, verapamil, iptakalim, calcium regulation. Inflammation/ROS-targeting: Nanoparticles, adenosine, nitric oxide, PDE inhibitors, antioxidants (4, 7).

Ischemic Conditioning: This involves repetitive short episodes of ischemia with intermittent reperfusion, showing protective effects in preclinical studies and potentially attenuating no-reflow. Most cardiology studies had information on the utility of ischemic postconditioning (4, 7).

Therapeutic Hypothermia: Induced cooling, particularly selective therapeutic cooling, has shown promise in protecting against no-reflow. In experimental models, more clinical evidence is needed. Hypothermia with therapeutic cooling and reducing ischemic volume and improving prognosis (4, 7).

## Discussion

The no-reflow phenomenon in acute ischemic stroke is a complex microvascular dysfunction that hinders functional recovery despite successful macrovascular recanalization. It is driven by microvascular occlusions, vascular remodeling, and the stalling of neutrophils. While various measurement techniques exist, a universally accepted method for quantifying no-reflow remains elusive. Molecular markers, particularly neutrophil-related indicators and inflammatory cytokines like IL-6, show promise in predicting and understanding this critical post-recanalization complication. The lack of consensus on diagnostic criteria and the limitations of current imaging techniques highlight the need for further research into effective, real-time diagnostic tools and integrated therapeutic strategies (7).
